# Editorial: Women's coping in various spheres in society: Challenges and opportunities

**DOI:** 10.3389/fpsyg.2022.1048370

**Published:** 2022-11-29

**Authors:** Orna Braun-Lewensohn, Claude-Hélène Mayer, Shir Daphna Tekoah

**Affiliations:** ^1^Conflict Management and Resolution Program, Ben-Gurion University of the Negev, Beersheba, Israel; ^2^Department of Industrial Psychology and People Management, University of Johannesburg, Johannesburg, South Africa; ^3^School of Social Work, Ashkelon Academic College, Ashqelon, Israel

**Keywords:** women, coping, challenges, opportunity, multidisciplinary

## Introduction

Women operate in various parts of society and in organizations, which have, in many societies, became extremely diverse in terms of culture, language, gender roles, competencies, and abilities. They experience challenges and stressful events throughout their lives (Braun-Lewensohn and Mayer, 2020). These challenges and events may affect their physical and mental health. This situation creates opportunities for each individual woman and for the development of a society (Mayer et al., 2018a).

Certain parts of society act in a gendered manner and gender biases often lead to gate-keeping, excluding women from certain spheres (De la Rey and Kottler, 1999; Mayer et al., 2018b). Women need to deal with a great deal of complexity on the local and global level. It has been argued that different core attributes might be helpful for dealing with complex and varied situations, such as curiosity, international experience, international management development, cross-cultural training, and intercultural sensitivity, as well as constructive development and psychological capital (O'Boyle Jr et al., 2011). Others have mentioned that the keys to managing a diverse and complex world are passion for diversity, intercultural empathy, and diplomacy (Javidan et al., 2016).

## The aim of this Research Topic

This special issue explores women in various situations and contexts. We aim to demonstrate, through the various articles, the different ways in which women encounter, experience, and cope with stressful and challenging environments and events in various spheres of different societies. The articles collected here demonstrate how these experiences contribute to women's distress, on the one hand, and to their growth, resilience, and leadership, on the other. These articles examine populations from many parts of the world, including China, India, Japan, Latin America, South Africa, Israel, Poland, and Spain, and describe studies based on different research methods. Ten of these studies were qualitative, seven were quantitative, and two were interpretive. The papers address themes such as employment (five articles), motherhood (five articles), armed conflict (three articles), and romantic partnership (two articles). Other articles in this issue concern attitudes toward lesbians, excluded young women, and the coping of an extraordinary woman.

## The contributions in this Research Topic

Below, we present a brief introduction to the articles that comprise this special issue organized according to the different highlighted themes.

### Employment

Gibbons et al. focus on higher education as a key driver of women's empowerment and claim that although women in Latin America well integrated as junior-level employees in academia, they are excluded from higher positions in this field. These researchers stress the importance of integrating women in this field and argue that doing so would promote the further involvement of women in education (i.e., providing role models for other women) and contribute to society in general.

Xue et al. address the career success of women in China, which affects the quality of their work and services. These researchers underscore the importance of this key factor for employment development, with a focus on the field of nursing. They suggest that managers should emphasize team stability and a sense of career success, to improve the resilience and satisfaction of female employees.

Jacobs and Barnard address the unique work environment of law enforcement. They highlight the experiences of women in this setting, which is highly aggressive, hostile, violent, and male-dominated. Despite the many challenges, they claim that women who work in this field are satisfied, resilient, and cope well. These authors report that these women feel authentic, present a mature sense of self, feel spiritual, and experience self-actualization.

Zinatsa and Donia Saurombe address a unique population within the workforce: tied migrants. They claim that, in addition to the regular challenges of the South African labor market, members of this population face additional barriers when they want to integrate in the labor market and that, for women, these challenges are even greater. These women face inequality due to their gender, race, and ethnicity and, therefore, they are not well integrated into the workforce and face both immobility and instability.

Kim investigated the value of female leadership in Japan, specifically its effect on diversity, an inclusion climate, and task-related positive attitudes of employees. She argues that there is a psychological mechanism by which female leadership contributes to positive attitudes of workers, which, in turn, facilitates a diversity climate and inclusion in the work sphere.

Studies addressing this topic in America, Asia, and Africa demonstrate the importance of this topic for women around the world. The authors focus on fields of employment in which women are still excluded and highlight the importance of women's leadership in various workplaces, to promote other women and to serve as role models for them. All of these papers note the importance of empowering women in different ways, including family support, religious faith, team stability, career success, and self-actualization.

### Motherhood

Two of the articles in this special issue address maternal and parental stress in China. Chen et al. studied families that include a child with special needs (i.e., autism) and, more specifically, the relationship between maternal sense of parenting efficacy and parental stress. They found that maternal sense of parenting efficacy predicted parental stress in such families and that family interaction moderated the relationship between maternal sense of parenting efficacy and parental stress. Dong et al., who also studied maternal parental stress in China, found that depression mediates the relationships between parenting stress and marital satisfaction. Additionally, loneliness is a significant factor for parenting stress and depression, but does not significantly affect marital satisfaction. They concluded that mothers who experience high levels of depressive symptoms while also experiencing parenting stress report low levels of marital satisfaction.

Two other studies on motherhood explore stress related to the COVID-19 pandemic. A Polish study by Pieta et al. investigated pregnant women, social support, and body image against the backdrop of this pandemic. They stress the importance of the mechanisms that women can use to gain more body satisfaction during pregnancy, which, in turn, can lead to the planning of more effective psychological interventions, especially against the background of this pandemic and its related psychological distress. An Israeli study by Shoshi et al. explored mothers of preterm infants and their experiences of COVID-19. All of the mothers in this study reported cumulative stress caused by the infant's health and COVID-19 stressors. Those mothers also feared infections and loneliness. On the other hand, they reported resources such as shared fate regarding the pandemic, improvements in their infant's condition, religious faith, emotional support from their partners, and support from professionals.

The last study on this topic, by Donath et al., took a unique look at Israeli women who are not sure whether they want to become mothers. Their findings question the typology of binary intersect and rigid classifications regarding women's reproductive decision-making. Those researchers found that while some women want to overcome their indecisiveness, others find that indecisiveness keeps options open for them and expands the boundaries of their autonomy.

### Armed conflict

The three papers that deal with armed conflict address armed conflict in Peru, Colombia, Israel and the USA. These three articles address different issues related to women in this context. The Israeli study, by Daphna-Tekoah et al., aims to explore and understand the combat experiences and challenges faced by American and Israeli women soldiers during and after their service. Those authors suggest that there is a need for a unique method to be used in research related to women veterans, in order for that research to be adequate. They argue that female veterans' voices will not be fully heard unless we allow them to be active participants in generating knowledge about themselves.

The Colombian study, by González-Castro et al., addresses war-related violence against women and explores its impact on post-traumatic stress and recovery among women who have been exposed to such violence. Their findings provide strong evidence that recovery following such events is affected by the ways in which these women are treated by society and by their families. The authors stress the importance of examining PTSD and recovery through the analysis of social processes, as opposed to employing only an individual focus, and note the importance of incorporating the findings of such analyses into intervention practices.

The last paper on this topic addresses issues of post-war growth after horrific events that occurred as part of the armed conflict in Peru. The authors, Tavara and Lykes, aimed to create opportunities for women who had experienced such events to reflect on those stressful times. The authors engaged in participatory action research, in which a group of women initiated economic and collective projects. They found that the wounds from this armed conflict have generated many different forms of silence, which have prevented these women from openly expressing how they feel. These authors highlight the limitations of interventions based exclusively on verbal communication and argue for action-based approaches that draw on the knowledge and practices of the affected population.

### Romantic partnership

Two papers from Israel address this topic from two different angles. The first paper, by Peichich-Aizen and Segal-Engelchin, explores the complexity inherent in relationships between a woman who does not have any children and a male widower who has young children. This article describes how many of these women feel that the deceased wife continues to be present in their partner's life and in their relationship, in a triangular system consisting of the woman, her partner, and his late wife.

Kook and Harel-Shalev examined a different sort of romantic partnership, focusing on the special issues that the practice of polygamy presents for minority women. They demonstrate the tension between the different mechanisms that the state uses to address such relationships and the mechanisms used by the women themselves. They suggest that the concept of ontological security can inform our understanding of the government's different motivations in cases related to minority women, violence, and the right to protection.

The last four papers deal with range of topics. A paper by Birger Sagiv et al. addresses social policies designed to help socially excluded young adult women. In a psychobiographical study, on Elizabeth Schuyler Hamilton Mayer presents the coping skills of one extraordinary woman. Chhajer et al. address safety concerns as key factors that discourage women from traveling. Finally, Tzur Peled et al. examine perceptions regarding the quality of perinatal care provided to lesbian women, in terms of the attitudes of nurses from different ethnic groups, subjective norms, behavioral intentions, and assessments of communication and relationships.

## Conclusions

### The way forward

In this special issue, we aim to explore women in various situations and contexts. Indeed, the 19 articles included in this collection demonstrate—through qualitative, quantitative, and interpretive methods—different ways in which women encounter, experience, and cope with stressful and challenging environments in various spheres of life in different societies around the world. We can conclude that in both Western societies and more culturally traditional societies, women face challenges and mostly cope well with them. Some of them are universal; women around the world still face conflicts regarding their workplace and regarding their motherhood and romantic partnerships, as well as challenges associated with armed conflicts. However, several challenges are unique to particular countries and/or cultures. One example, which is common in several developing countries and societies, is the challenge of studying and developing through higher education. Another more unique challenge, which exemplifies the conflict faced by members of a traditional culture within a Western country, is the challenge of polygamy and the way the state deals with that issue.

Many researchers and organizations around the world are currently working to promote and empower women. Still much work remains to be done, in order to close gaps and create a universal egalitarian society. Policymakers should become aware of the different studies collected here, which we hope will contribute to research-based, theory-driven policies and prevention and intervention programs to promote safe and equal treatment of women. Efforts made toward these goals will help women to better adjust and cope in different spheres.

## Author contributions

All authors listed have made a substantial, direct, and intellectual contribution to the work and approved it for publication.

## Conflict of interest

The authors declare that the research was conducted in the absence of any commercial or financial relationships that could be construed as a potential conflict of interest.

## Publisher's note

All claims expressed in this article are solely those of the authors and do not necessarily represent those of their affiliated organizations, or those of the publisher, the editors and the reviewers. Any product that may be evaluated in this article, or claim that may be made by its manufacturer, is not guaranteed or endorsed by the publisher.
